# Core-Penumbra Hyperacute Ischemic Stroke Dataset

**DOI:** 10.1038/s41597-025-05000-0

**Published:** 2025-04-29

**Authors:** D. Umerenkov, S. Kudin, M. Peksheva, D. Pavlov

**Affiliations:** 1 AIRI, Moscow, Russia; 2 Sber AI Lab, Moscow, Russia; 3 Hospital 40 of the St Petersburg Resort district, St Petersburg, Russia

**Keywords:** Stroke, Stroke

## Abstract

We introduce the CPAISD: Core-Penumbra hyperAcute Ischemic Stroke Dataset, aimed at enhancing the early detection of ischemic stroke using Non-Contrast Computed Tomography (NCCT) scans. Hyperacute ischemic stroke is the earliest phase of stroke, characterized by subtle or absent findings on NCCT scans, which complicates timely diagnosis. The dataset provides a collection of segmented NCCT images, including annotations of ischemic core and penumbra regions, critical for developing machine learning models for rapid stroke identification and assessment. By offering a carefully collected and annotated dataset, we aim to facilitate the development of advanced diagnostic tools, contributing to improved patient care and outcomes in stroke management. Our dataset uniquely focuses on the hyperacute phase, where NCCT scans typically show minimal or no signs of stroke, and includes a baseline model demonstrating its utility.

## Background & Summary

Stroke, a leading cause of long-term disability and the second leading cause of death globally^1^, presents a significant challenge in medical imaging and diagnosis. The ability to rapidly and accurately diagnose stroke and determine the affected volumes is paramount in selecting appropriate treatment strategies to mitigate the devastating consequences of this condition. The traditional approach employs a multi-stage imaging protocol, beginning with a Native (Non-Contrast) CT scan of the brain, followed by more specialized scans such as CT Angiography (CTA) of the Brachiocephalic Arteries and CT Perfusion (CTP) Imaging of the brain^2^. Additionally, Magnetic Resonance Imaging (MRI) is a reliable diagnostic tool for stroke. MRI provides detailed brain imaging crucial for precise stroke assessment, but limited availability restricts its use primarily to larger hospitals. Although comprehensive, the multi-stage CT or MRI imaging process is time-consuming and often unavailable, limiting its effectiveness in urgent hyperacute stroke cases.

The initial phase of an ischemic stroke is crucial for diagnosis, yet it frequently poses significant challenges. Native CT scans, typically the first imaging choice, frequently miss subtle early signs of ischemia, such as the blurring of the boundary between grey and white matter or the flattening of brain sulci. This absence of detectable changes can significantly delay the diagnosis and initiation of treatment, negatively impacting the recovery prospects of patients. While later imaging stages like CT Angiography and CT Perfusion are critical for assessing the presence of major arterial blockages and the extent of brain tissue damage, they require more time to perform and may not be readily accessible, particularly in resource-limited or rural healthcare settings.

Imaging models capable of reliably segmenting stroke areas using only NCCT scans have significant implications for the management of stroke patients. Most importantly, they enable faster diagnosis and treatment, which is critical in time-sensitive situations. Their use expands access to stroke diagnostics, especially in regions where advanced imaging technologies are less available. Furthermore, NCCT scans are less invasive compared to contrast-based imaging, as they do not require contrast administration. These models can also improve triage and facilitate quicker transfer decisions, allowing patients to receive timely and appropriate care, an advantage supported by recent evidence demonstrating shorter treatment times with AI-assisted platforms^3^.

The availability of open datasets containing segmented images of acute ischemic stroke is crucial for the development and validation of stroke detection models using NCCT scans. These datasets serve as a critical resource for researchers and developers, allowing them to train and refine algorithms capable of identifying and segmenting ischemic areas with high precision. The benefits of such datasets are manifold:

- **Enhanced Model Training**: Open access to segmented NCCT images provides a diverse data pool for training machine learning models, ensuring they learn from a wide range of stroke presentations.
- **Improved Model Validation and Benchmarking**: These datasets allow for rigorous testing and comparison of different models, ensuring their reliability and accuracy in real-world clinical settings.
- **Promoting Equity in Healthcare**: Open datasets ensure that advancements in stroke detection are not limited to well-resourced institutions but are available for broader application, including in underserved regions.
- **Reducing Development Costs and Time**: Access to pre-segmented and curated datasets reduces the resources required for data collection and preparation, expediting the development of effective diagnostic tools.

In this context, we present a novel dataset comprising NCCT scans along with corresponding ischemic core and penumbra segmentation masks for patients with early ischemic stroke. A distinctive aspect of this dataset is that, for most cases, initial NCCT scans showed no visible ischemic changes, even to experienced radiologists.

This dataset offers a unique opportunity to explore the potential of advanced imaging techniques in augmenting the diagnostic capabilities of NCCT scans in early stroke stages. The inclusion of markup based on CTA and CT Perfusion data provides a rich source of information for developing and testing predictive models.

We also provide a baseline model developed to predict the findings of the more advanced CTA and CT Perfusion stages using only the data from the NCCT scans. While this model has not been extensively optimised it demonstrates the capabilities of AI models to find ischemic stroke areas that are hard or impossible to find for human expert.

### Related work - Models

Recent advances in medical imaging and machine learning have significantly enhanced the early identification and assessment of acute ischemic stroke using non-contrast computed tomography. Radiomics-based approaches, for instance, have demonstrated great promise in detecting ischemic changes that are imperceptible to radiologists, as evidenced by a patch classification model attaining high accuracy on NCCT scans^4^. Another notable development is EIS-Net, a multi-task learning method capable of segmenting early infarct regions while simultaneously computing the Alberta Stroke Program Early CT Score (ASPECTS)^5^.

Building on these successes, the nnU-Net framework was recently evaluated for its efficacy in segmenting early ischemic changes in patients with acute ischemic stroke, showing encouraging results^6^. Meanwhile, a neural network-based technique for automated ASPECTS calculation demonstrated notable sensitivity and accuracy in analyzing AIS patient CT scans^7^. In a related vein, a random forest classifier leveraging texture features has also been proposed to automate ASPECTS scoring, further underscoring the potential of machine learning for guiding stroke diagnosis and management^8^.

### Related work - Datasets

Several datasets have been developed to study acute ischemic stroke, encompassing both MRI and NCCT imaging techniques. Compared to a number of MRI-focused datasets, there are only two NCCT datasets for acute ischemic stroke. The first, AISD^9^, comprises 397 NCCT scans of acute ischemic stroke, captured within 24 hours of symptom onset. These patients also underwent diffusion-weighted MRI within the same timeframe. The second NCCT dataset, APIS^10^, introduces a paired CT-MRI dataset meticulously built for ischemic stroke segmentation. It includes 96 studies from patients exhibiting stroke symptoms, divided into control (n=10) and ischemic stroke (n=86) groups.

## Methods

The dataset used in this study originated from the Regional Vascular Center of the State Budgetary Healthcare Institution No40 of St. Petersburg. A total of 135 patients were included, with data collected between 2017 and 2020.

Two of the authors had direct clinical affiliations with Hospital 40 of the St. Petersburg Resort district and obtained authorization from the institutional ethics board to access this dataset. Following this approval, data were shared among all authors involved in the study. The use of this data specifically for training stroke prediction models and for its subsequent sharing with the broader scientific community was explicitly authorized by the ethics board of Hospital 40 of the St. Petersburg Resort district (decision dated 29.02.2024, protocol number 247). A waiver of consent was granted by the ethics board due to the complete anonymization of the dataset, ensuring that data files did not contain any identifying patient information.

All patients underwent comprehensive computed tomography (CT) evaluations comprising native scanning, CT angiography, and CT perfusion. Patients were admitted during the hyperacute phase of ischemic stroke, specifically within 24 hours from the onset of symptoms. However, precise timing from symptom onset was frequently unavailable, as a considerable number of patients were either discovered unconscious by others or awoke exhibiting stroke symptoms. Among the selected patients, there were 25 fatal outcomes. Additionally, 23 patients were excluded from further processing due to reasons such as damaged DICOM data and motion artifacts. For subsequent processing, 112 patients were selected. Of these, 40 did not undergo subsequent native CT scanning on the first day but had CT or MRI control on the second or third day; 41 patients were selected for endovascular treatment.

Out of the 112 selected patients:

- 1 patient with MCA stroke at both hemispheres, contains ischemic core.
- 2 patients with ICA stroke at both hemispheres, contains ischemic core (all lethal).
- 4 patients with right ICA stroke, 3 patients contains ischemic core.
- 38 patients with right MCA stroke, 1 patient had lacunar stroke, 28 patients contains ischemic core.
- 1 patient with right ACA stroke, without ischemic core.
- 5 patients with left ICA stroke, all contains ischemic core.
- 54 patients with left MCA stroke, 2 patients has lacunar stroke, 37 patients contains ischemic core.
- 7 patients with stroke at basilar artery territory, 6 patients contains ischemic core.

The scanning protocols used in the studies of this dataset are presented in Table 1. All scans were performed on either a GE Revolution EVO or a Siemens Somatom Emotion scanner. The perfusion maps where generated using Syngo via VB10B by Siemens.

**Table 1:** CT Scanning Protocols for GE Revolution EVO and Siemens Somatom Emotion.

Parameter	GE Revolution EVO	Siemens Somatom Emotion
Non-Contrast CT (NCCT)		
kV	120	130
mA or Noise Index	Noise Index 7 (ODM)	240 mA (no automodulation)
Rotation time	0.5 s	1 s
Coverage	16—14 cm (axial)	16 cm
Slice thickness	1.25 mm	2 mm
Pitch	0.96	0.9
Collimation	1.2	1.2
Acquisition	Not specified	16 × 1.2 mm
Kernel	Head	Head H31 (medium smooth)
Bowtie filter	Standard	Not specified
Reconstruction	ASIR-V 40%	—
Field of view (FOV)	320 × 320 mm	193 × 193 mm
**CT Perfusion (CTP)**		
kV	80	80
mA	180 (fixed)	220 (no automodulation)
Rotation time	0.4 s	1 s
Coverage	8 cm (shuttle mode)	16 × 1.2 mm (9.6 mm slice)
Kernel	Head	Angio head
Bowtie filter	Standard	Not specified
Scan time	17 passes over 43.8 + 1.8 s	40 s
Reconstruction	ASIR-V 10%	—
Field of view (FOV)	800 × 800 mm	200 × 200 mm
**Contrast Injection**		
Flow rate	6 mL/s	8 mL/s
Contrast	45 mL Omnipaque 350	50 mL Ultravist 370
Saline flush	30 mL	30 mL

Subsequently, a radiologist with 10 years of experience performed the markup of the native scanning series across all sections, comparing with CT perfusion maps, and delineated areas of penumbra and core (one within the other). No additional software support tools (e.g., edge detection) were employed; segmentation was manually performed using standard Horos software settings, guided solely by anatomical landmarks such as ridges, gyri, and sulci.

The use of this data for training of stroke prediction models and the release of this data to the community was authorised by the decision of the ethics board of Hospital 40 of the St Petersburg Resort district on 29.02.2024, protocol number 247.

## Data Records

The dataset is available at Zenodo, reference number 10892316^11^. The dataset includes 112 non-contrast cranial CT scans of hyperacute stroke patients, each featuring manually annotated ischemic core and penumbra regions across individual slices.

The dataset is divided into three parts: **Training** (92 studies, 8,376 slices), **Validation** (10 studies, 980 slices), and **Testing** (10 studies, 809 slices). Overall, there are 10,165 slices across 112 studies, with a mean of 91 slices per study (IQR 80-100).

The data have been anonymized using the Kitware DicomAnonymizer^12^ under standard settings, except that the following DICOM fields are preserved for demographic and device-related analyses:

- (0 × 0010, 0 × 0040) - Patient’s Sex
- (0 × 0010, 0 × 1010) - Patient’s Age
- (0 × 0008, 0 × 0070) - Manufacturer
- (0 × 0008, 0 × 1090) - Manufacturer’s Model Name

The dataset is organized into three top-level directories named train, val, and test. Each top-level directory contains multiple study folders. Each study folder includes:

- metadata.json - study-level metadata (e.g., manufacturer, model, age, sex, dsa, etc.).
- Slice subfolders, each containing:
  - raw.dcm - original DICOM file.
  - image.npz - slice data in .npz (NumPy) format.
  - mask.npz - corresponding segmentation mask in .npz format.
  - metadata.json - slice-level metadata.

At the root of the entire dataset, a metadata.json file captures:

- generation_params - parameters used for dataset creation (including test_size and val_size).
- stats - statistical data summarizing pixel-value distributions and the number of studies/slices in each split.

The metadata.json for each study contains fields for manufacturer, model, device, age, sex, dsa, nihss, time, and lethality. If a value is unknown, it is reported as -unknown.- For convenience, a summary.csv provides the same study-level information, along with name (study identifier) and part (which split the study belongs to).

## Technical Validation

All the data was manually validated by cross-checking the DICOM tags with separate records containing lethality information to ensure consistency.

We used the CPAISD dataset to train a baseline core and penumbra segmentation model. The baseline model is a segmentation network of the FPN^13^ architecture with an efficientnet-b0^14^ backbone, segmenting a single-channel input image into three classes. The network receives a single slice as input and outputs a three-channel mask, with class 0 representing the background, class 1 the stroke core, and class 2 the penumbra.

The network was trained using the Adam optimizer, with DiceLoss as the loss function, computed only for the stroke core and penumbra classes. The learning rate scheduler used was ReduceLROnPlateau. We trained the model using a batch size of 32 and an initial learning rate of 0.006. Images across all dataset splits (training, validation, and testing) were normalized using the mean and standard deviation derived from the training set. Prospective images for inference were likewise normalized using the same training-set statistics. Data augmentation included horizontal flipping and random rotations within 10 degrees.

A previously published model for predicting stroke core and penumbra from perfusion data^15^ reported 3D Dice scores of approximately 0.86 for the core and 0.83 for the penumbra (for details on the metric definition, please see the referenced study.) Our method, relying solely on NCCT scans, resulted in lower performance due to the limited diagnostic information inherent in non-contrast imaging. Our model achieved average 3D Dice scores of 0.21 (core) and 0.35 (penumbra) on the test portion of our dataset.

In addition, we computed 3D sensitivity (mean 0.579, std 0.159), specificity (mean 0.857, std 0.115), negative predictive value (mean 0.778, std 0.089), and positive predictive value (mean 0.611, std 0.199) on the test set.

**Fig. 1:**
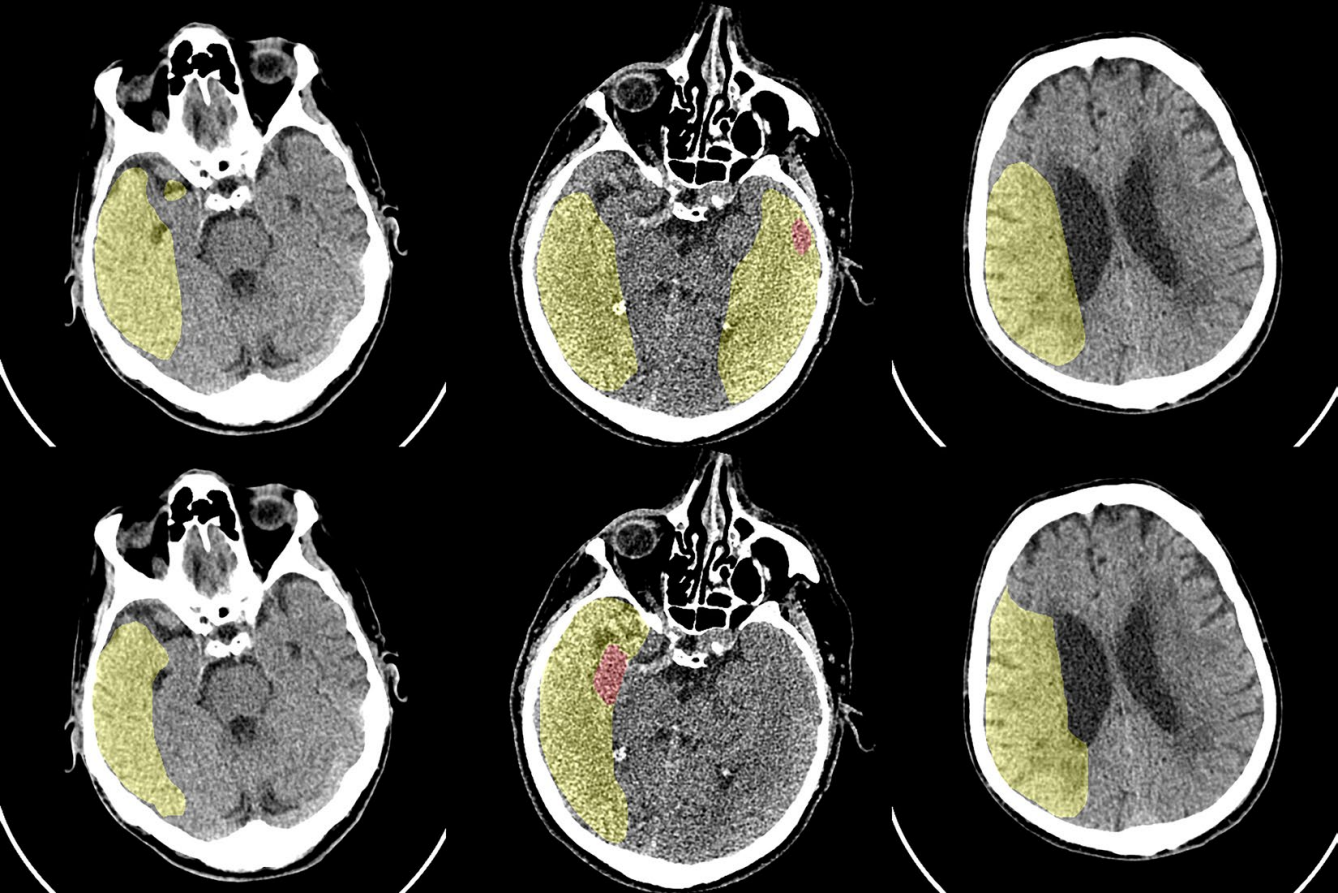
Baseline model prefictions. Predictions on the top row, ground truth in bottom. Core is shown in red, penumbra in yellow.

The example of baseline model prediction and the ground truth is shown in Figure 1.

## Data Availability

The baseline model code and weights can be accessed at https://github.com/sb-ai-lab/early_hyperacute_stroke_dataset.
